# *Megastigmus* seed wasp damage on native *Schinus terebinthifolia* drupes in ecological restoration area in Brazil

**DOI:** 10.1038/s41598-019-39129-x

**Published:** 2019-02-21

**Authors:** Thaís Carneiro Ghiotto, Marcelle Cristine do Nascimento Prado, Graziella Kurpjuweit Fischer Giuliani, Wagner de Souza Tavares, Marcus Vinicius Masson, Julio César Guerreiro, Evandro Pereira Prado, Amélia Guimarães Carvalho, Carlos Frederico Wilcken, José Cola Zanuncio, Pedro José Ferreira-Filho

**Affiliations:** 1 Departamento de Ciências Ambientais, Universidade Federal de São Carlos, 18052-780 Sorocaba, São Paulo Brazil; 2 Bahia Specialty Cellulose/Copener Florestal Ltda., 48030-480 Alagoinhas, Bahia Brazil; 3 Departamento de Agronomia, Centro de Ciências Biológicas, Universidade Estadual de Maringá, 87507-190 Umuarama, Paraná Brazil; 4 Faculdade de Ciências Agrárias e Tecnológicas, Universidade Estadual Paulista “Júlio de Mesquita Filho”, 17900-000 Dracena, São Paulo Brazil; 5 Departamento de Engenharia Florestal, Universidade Federal de Viçosa, 36570-900 Viçosa, Minas Gerais Brazil; 6 Departamento de Proteção Vegetal, Universidade Estadual Paulista “Júlio de Mesquita Filho”, 18603-970 Botucatu, São Paulo Brazil; 7 Departamento de Entomologia/BIOAGRO, Universidade Federal de Viçosa, 36570-900 Viçosa, Minas Gerais Brazil

## Abstract

*Megastigmus transvaalensis* Hussey (Hymenoptera: Torymidae) parasitizes drupes of *Rhus* genus plants in Africa and *Schinus* (Anacardiaceae) in South America. This exotic wasp damages *Schinus terebinthifolia* Raddi drupes in native forests and ecological restoration areas in Brazil. The objective of the present study was to investigate the precipitation, temperature and relative humidity effects on *M*. *transvaalensis* flight activity, and to determine the parasitism rate and sex ratio of this wasp on *S*. *terebinthifolia* plants. The study was conducted with yellow sticky traps and *S*. *terebinthifolia* drupes collected in an ecological restoration area, from August 2014 to September 2015, in the Sorocaba municipality, São Paulo state, Brazil. *Megastigmus transvaalensis* populations were negatively correlated with maximum and minimum temperatures and precipitation, with population peaks at the end of May 2015, with 927 insects per evaluation (48.8 adults per trap). The *M*. *transvaalensis* sex ratio was higher in the laboratory (0.42) than in the field (0.08). The parasitism rate of *S*. *terebinthifolia* drupes by *M*. *transvaalensis* ranged from zero to 36.3% under natural environmental conditions. *Megastigmus transvaalensis* can be monitored with yellow sticky traps. Damage by *M*. *transvaalensis* in *S*. *terebinthifolia* drupes may decrease the germination of the seeds and the establishment of this plant in native and restoration ecological areas.

## Introduction

It is important to determine the geographic origin of the organisms to assess their impact on the ecosystems^1,2^. *Schinus terebinthifolia* Raddi, 1820 (Sapindales: Anacardiaceae), native to Argentina, Brazil and Paraguay^3^ presents pioneering behavior, attractiveness for avifauna, good development in low fertility soils and it is used in land recovering of degraded areas in Brazil^4^. This plant adapts to different environmental conditions with high competitiveness and easy cultivation^5^.

*Schinus terebinthifolia* occurs from Pernambuco state to southern Brazil on sandy and clayey soils and is also reported in temperate regions^6^. Its drupes, collected in native areas, are an income source in the Espírito Santo state, Brazil^7^. This plant was introduced into more than 20 countries, including Australia, Bermuda, Fiji, Mauritius, Micronesia, New Caledonia, Reunion Island, South Africa, and Tahiti^3^ and the Bahamas and Virgin Islands in the Caribean^8^ with fruits used for ornamental purposes and in the spice trade^9,10^. In 1980, *S*. *terebinthifolia* was introduced into Florida, USA, proving to be highly invasive^11^ and spreading to Arizona, California, Hawaii, and Texas^8^. *Schinus terebinthifolia* has an allelopathic effect, preventing seed germination of native species^12–14^ and causing environmental impacts^11^.

In Florida, the Department of Agriculture and Consumer Services classified *S*. *terebinthifolia* as very harmful and the USA has banned the sale of this plant^8,11^. The uncontrolled increase of the area occupied by *S*. *terebinthifolia* led to studies with the parasitoids of their drupes^15,16^ as candidates for the biological control of this plant, but none were effective in the USA^17^.

In 1988, *Megastigmus transvaalensis* Hussey, 1956 (Hymenoptera: Torymidae), unknown in the USA, was recovered and reared from *S*. *terebinthifolia* drupes collected in Palm Beach County^8^. This phytophagous wasp, native to South Africa, may reduce *S*. *terebinthifolia* germination^18^.

*Megastigmus transvaalensis* parasitizes drupes of *Rhus angustifolia* L. and *Rhus laevigata* L. (Sapindales: Anacardeaceae) and *S*. *terebinthifolia* and *Schinus molle* L. (Sapindales: Anacardeaceae) in South Africa^19^, *Schinus polygamus* (Cav.) Cabrera in Chile^20^ and *S*. *terebinthifolia* in Paraná^21^ and São Paulo state, Brazil^22^. The *M*. *transvaalensis* embryonic period is four to five days with its larval stage lasting from 20 to 25 d^23^ with pupation inside the fruits, where it can remain in diapause for months with its adults emerging during *S*. *terebinthifolia* flowering and fruit formation stages^24^.

In Brazil, unlike the USA, *S*. *terebinthifolia* is important mainly in riparian forest recovery and dune stability programs and projects^25^, where *M*. *transvaalensis* may limit the development of this plant^22^.

Integrated pest management depends on insect population monitoring, mainly peaks and relationships with abiotic factors^26,27^. Capture with traps favors abundance, sampling, population dynamics and monitoring studies of insect pests^28–30^.

The objectives of this study were to investigate the precipitation, temperature and relative humidity effects on *M*. *transvaalensis* flight activity, and to determine the parasitism rate and sex ratio of this wasp on *S*. *terebinthifolia* plants.

## Results

### Numbers of *M*. *transvaalensis* adults

The number of *M*. *transvaalensis* adults caught in the sticky traps, varied with the seasons, with 3,415 insects captured, being 279 females and 3,136 males. The number of *M*. *transvaalensis* adults during the summer, when the rains were more constant (690 mm) and the minimum and maximum temperatures remained around 20.5 and 26 °C, was about two individuals per trap. This number increased from the 18^th^ evaluation (May 6, 2015), during the fall, when rains were less intense (138 mm) and the minimum and maximum temperatures were 17 and 22 °C, respectively. The *M*. *transvaalensis* population peak of 927 insects in the 19^th^ evaluation (May 20, 2015) in autumn, coincided with lower precipitation (13 mm) and temperature (20.1 °C). *Megastigmus transvaalensis* were not captured in the third (October 10, 2014) and 14^th^ (March 11, 2015) evaluations in 19 sticky traps on *S*. *terebinthifolia* plants.

**Figure 1:**
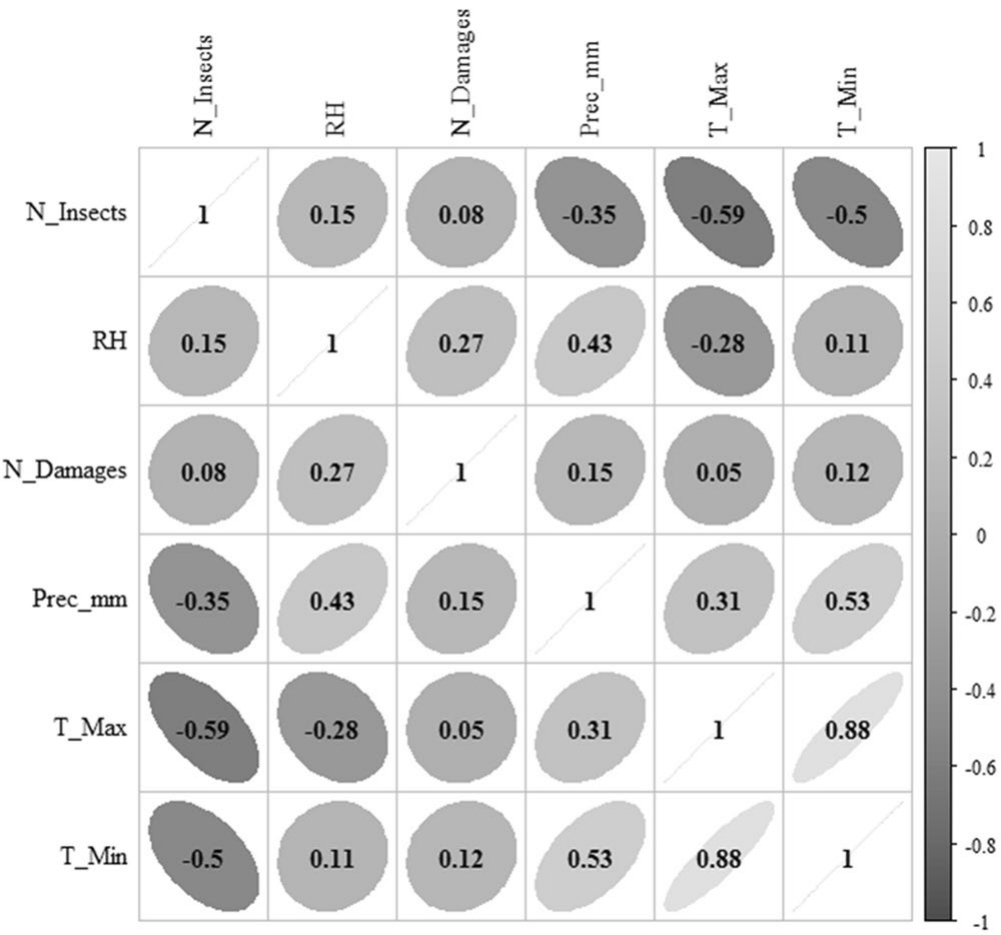
Linear correlation matrix between the response variable insects number (N_insects) and the explanatory variables minimum temperature (T_Min), maximum temperature (T_Max), relative humidity (RH), accumulated precipitation (Prec_mm) and damaged drupes (N_damages). Sorocaba, São Paulo state, Brazil. August 29, 2014 to September 9, 2015. Captions: N_Insects = insects number; RH = relative humidity; N_Damages = damages number; Prec_mm = precipitation; T_Min = minimum temperature; and T_Max = maximum temperature.

The correlation matrix presenting the transformed data showed a linear inverse relationship between the variable number of insects (N_Insects) and explanatory variables, minimum (T_Min) (r = −0.50) and maximum (T_Max) (r = −0.59) temperatures, precipitation (r = −0.35) and low correlation with relative humidity (r = 0.15) and drupes damaged (r = 0.08) (Fig. 1). Differences between the independent and dependent variables presented more significant effects from each other. Minimum (*P* = 0.0013) and maximum (*P* = 0.0034) temperatures influenced the dependent variables.

The distribution of the database was normal with a value of W = 0.4835 showing that the samples were derived from the same distribution, with non-significant results (*P* = 0.9438) and indicating a good fit for the model. The database was compatible and could be used to interpret and discuss the variables. The hypothesis that population variability is similar (variance homogeneity), that is, that there are differences between the *M*. *transvaalensis* populations, was rejected.

**Figure 2:**
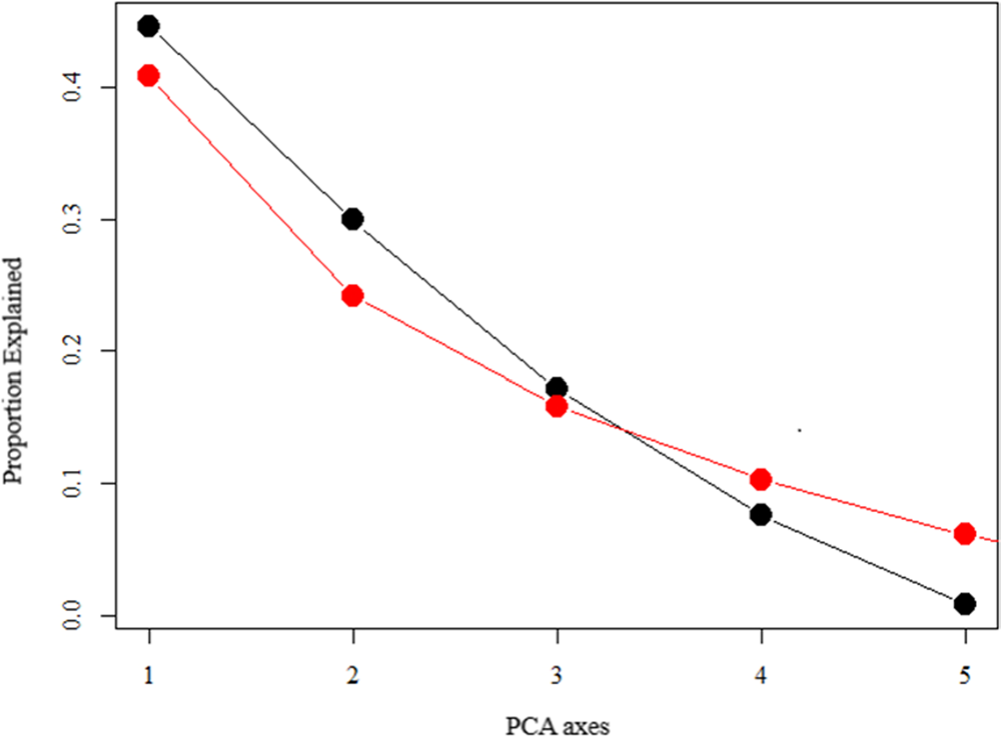
Broken-stick model for the response variable insects number (N_insects) and explanatory variables minimum temperature (T_Min), maximum temperature (T_Max), relative humidity (RH), accumulated precipitation (Prec_mm) and damaged drupes N_Damages) of principal component analysis (PCA).

**Figure 3:**
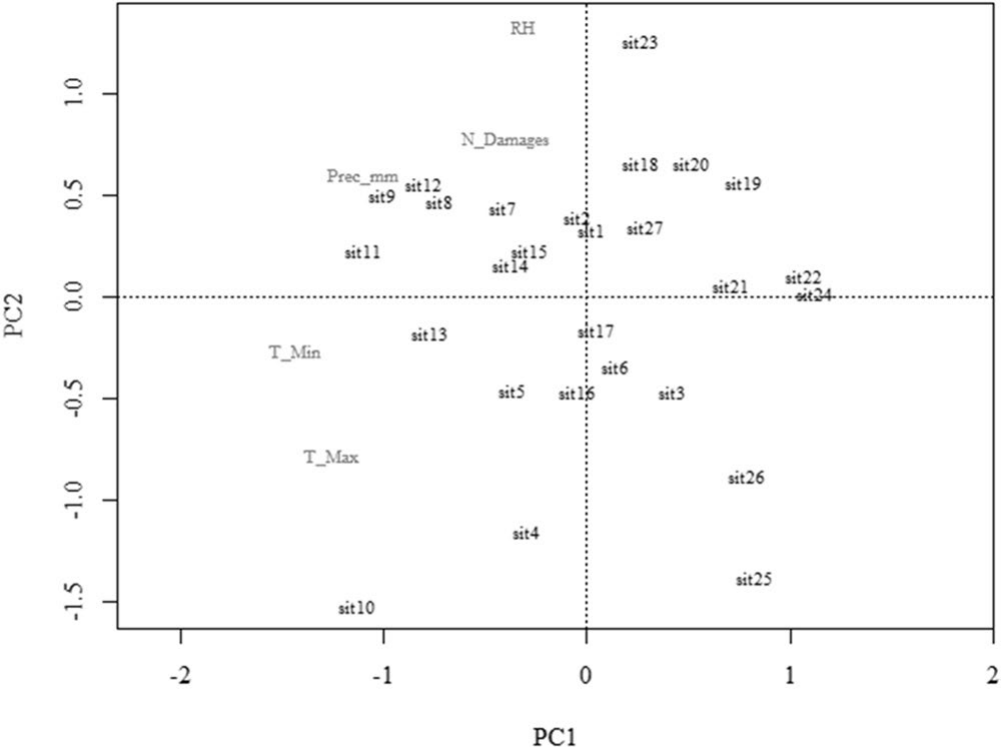
Principal components of main component analysis (PCA) containing the explanatory variables minimum temperature (T_Min), maximum temperature (T_Max), relative humidity (RH), accumulated precipitation (Prec_mm) and damaged drupes (N_Damages). Sorocaba, São Paulo state, Brazil. August 29, 2014 to September 9, 2015. Captions: sit = evaluations number.

**Figure 4:**
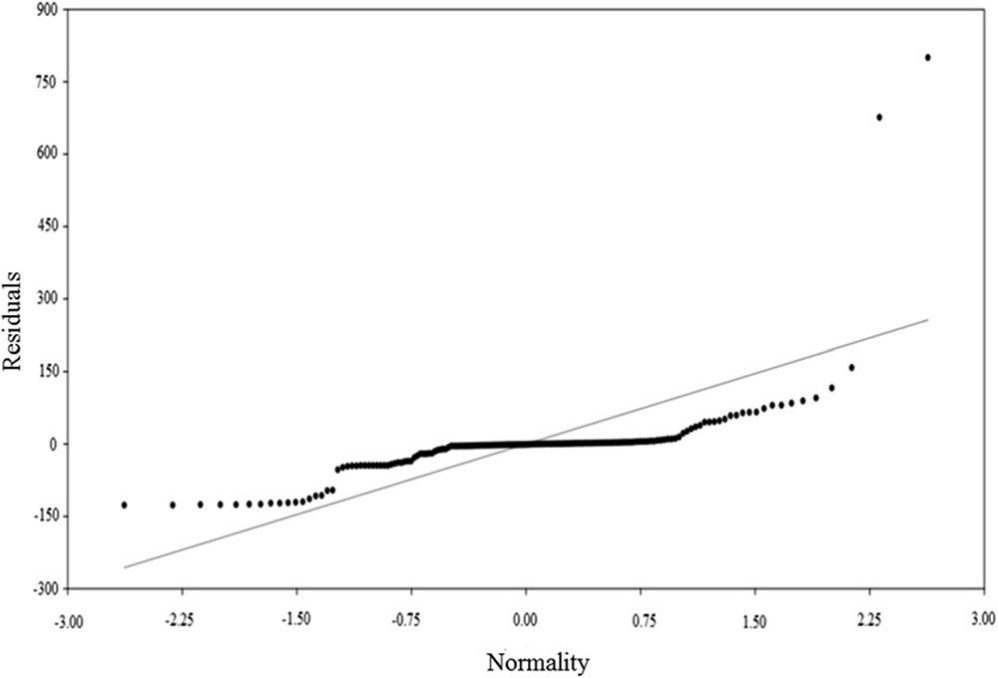
Residual analysis obtained from the scores related to the simple linear regression equation, characterized by the comparison between the values of the response variable insects number (N_insects) of *Megastigmus transvaalensis* (Hymenoptera: Torymidae) and the explanatory variables minimum temperature (T_Min), temperature (T_Max), relative humidity (RH), accumulated precipitation (Prec_mm) and *Schinus terebinthifolia* damaged drupes (Anacardiaceae) (N_Damage).

The main component analysis showed a relationship with the response variable and can be evaluated with the PC1 and PC2 axes, since they have an eigenvalue of more than one, 43.93 and 26.32%, respectively, of the data set variation (Fig. 2). The PCA retained all factors with eigenvalues greater than 1 (Table 1), showing that the variables belong to certain axes. Broken-stick axes 1 and 2 explain most of the variability, with a steep decrease (14.51%) from axis 3. The variability ratio was low for axes 4 (8.72%), 5 (5.93%) and 6 (0.06%) (Table 1 and Fig. 2). The correlation between the variables and the PC 1 axis was high and inversely proportional to the minimum (r = −0.93) and maximum (r = −0.89) temperatures, moderate for precipitation (r = −0.65) and low for drupes damage (r = −0.13) and relative humidity (r = −0.03) (Fig. 3). The PC1 axis showed that the variable number of insects (N_Insects) and the explanatory minimum (T_min) and maximum (T_max) temperatures and precipitation (Prec_mm) are more strongly related to the number of *M*. *transvaalensis* individuals captured. The adjusted coefficient of determination (R^2^ adj. = 0.2882; n = 27) showed how the regression analysis line fits the data set (Fig. 4). Low R^2^ adj. values do not always mean bad models, since the relationship between the analyzed variables and the normality of the data and the value of F (F = 31.062) should be considered. This is necessary to determine the linear relationship between the dependent and independent (Table 2) variables. The PC 2 axis was not significant (*P* = 0.8149).

**Table 1:** Table produced with scores resulting from principal component analysis (PCA) containing the eigenvalues, proportion explained and cumulative proportion by main component of the variables.

	Principal Component Analysis (PCA)				
	PC1	PC2	PC3	PC4	PC5
Eigenvalue	2.2303	1.4941	0.8575	0.3787	0.0394
Proportion explained	0.4461	0.2988	0.1715	0.07574	0.0078
Cumulative proportion	0.4461	0.7449	0.9164	0.9921	1.0000

**Table 2:** Linear regression model of Adrien-Marie Legendre (1805) from the principal components analysis (PCA) with the PC1 and PC2 axis for the response variable *Megastigmus transvaalensis* (Hymenoptera: Torymidae) insects number (N_Insects).

Response variable	N_insects	R^2^ adj.	F	df1, df2	*p*-valor
Insect numbers	27	0.2882	31.062	5.21	0.0052

The number of *M*. *transvaalensis* individuals captured in the field varied with temperature and precipitation. Population peaks of this insect were higher from May to August 2015, mainly in late May and early June, with inversely proportional correlation with minimum and maximum temperatures (transition from autumn to winter) and precipitation.

Relative humidity did not affect *M*. *transvaalensis* population peaks in May or June 2015, that is, did not show any association with the variable response relative humidity (*P* = 0.1885). The drupe damage explanatory variable showed no relation with the number of insects captured (*P* = 0.0506).

### Parasitism rate

**Figure 5:**
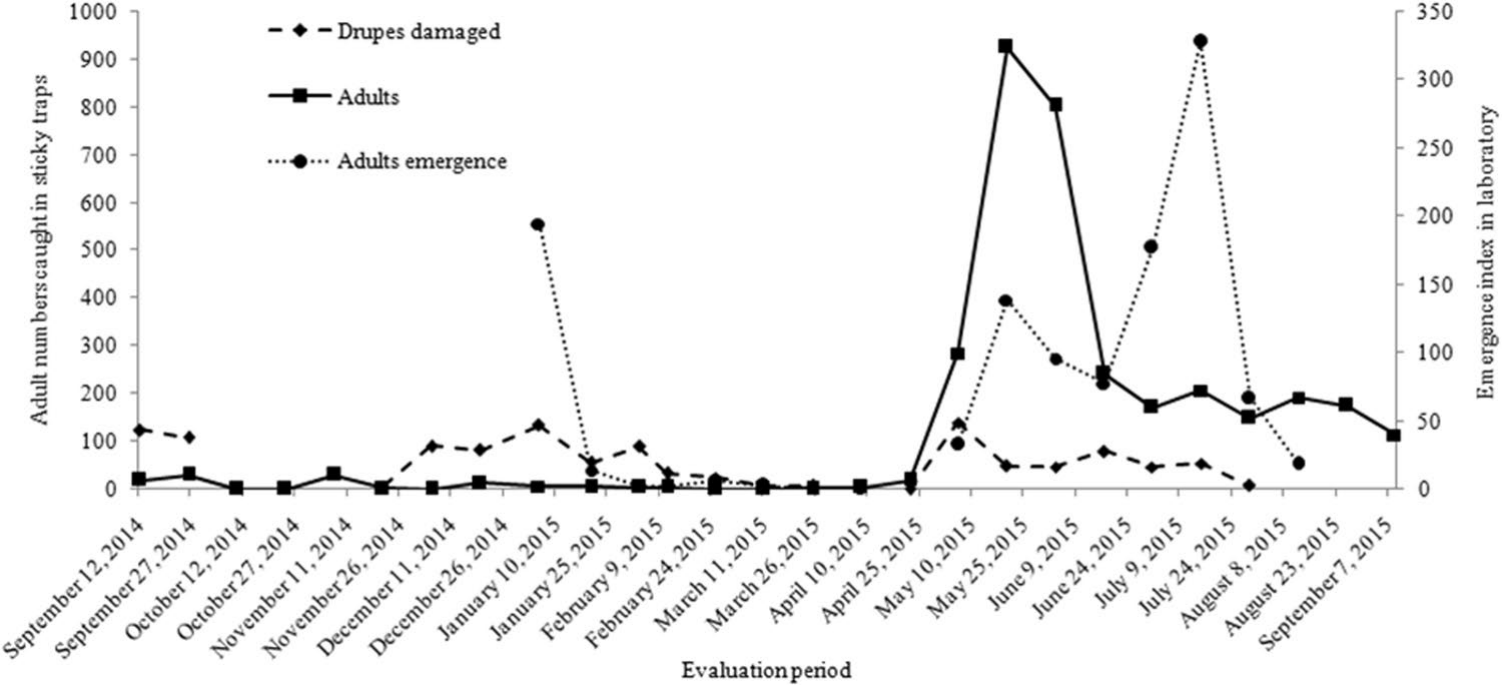
Drupes damaged, total of adults caught in yellow sticky traps and of adults emerged of *Megastigmus transvaalensis* (Hymenoptera: Torymidae) from *Schinus terebinthifolia* (Anacardiaceae) drupes. Sorocaba, São Paulo state, Brazil. August 29, 2014 to September 9, 2015.

The damaged *S*. *terebinthifolia* drupe numbers were higher in September (15.6%), November (36.3%) and December (19.3%) in 2014 (Fig. 5). Differences in the number of drupes produced per plant may explain variation in the damage in those parasitized by *M*. *transvaalensis* (F = 33.75, df = 36.54, *P* = 1.187e-06).

### Sex ratio

**Figure 6:**
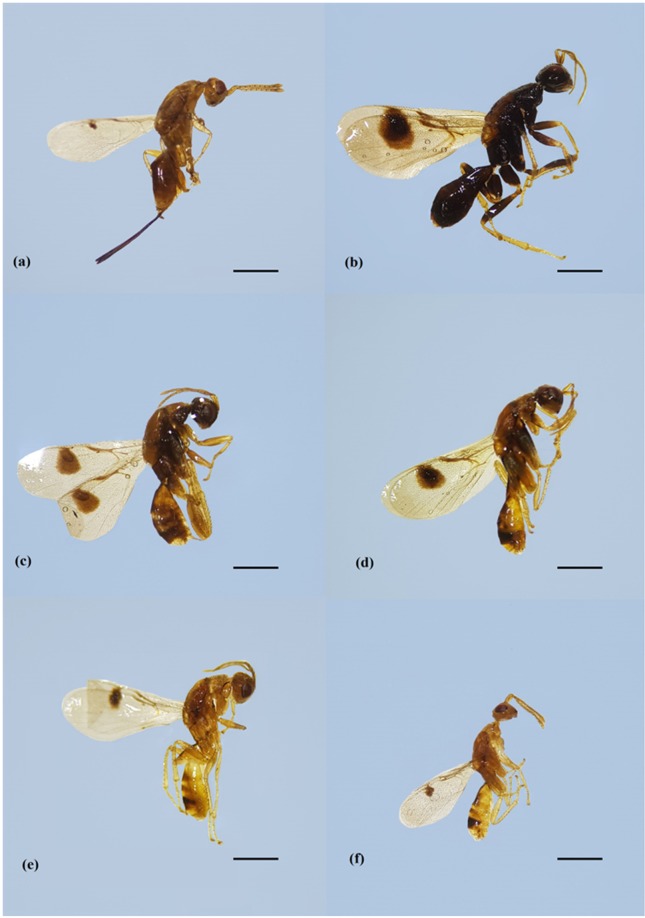
*Megastigmus transvaalensis* (Hymenoptera: Torymidae) female and male phenotypic variation (**b**–**f**) emerged from *Schinus terebinthifolia* (Anacardiaceae) drupes in laboratory. Sorocaba, São Paulo state, Brazil. August 29, 2014 to September 9, 2015. Scale bars = 1 mm.

The *M*. *transvaalensis* adult numbers emerged in the laboratory was 1,161, with 39.6 males and 28.6 females per evaluation. In the field, the *M*. *transvaalensis* adult numbers caught in the sticky traps was 3,415, with 116.4 males and 10.0 females per evaluation. The sex ratio in the laboratory was higher (0.42) than in the field (0.08) (Table 3). The number of adults caught in the field traps was similar between collections (F = 1.973, df = 37.15, *P* = 0.1684), but it varied in the laboratory (F = 7.015, df = 15.09, *P* = 0.0181) (Fig. 5). *Megastigmus transvaalensis* males showed phenotypic variation (Fig. 6a–f).

**Table 3:** Mean number of females and males and *Megastigmus transvaalensis* (Hymenoptera: Torymidae) sex ratio in laboratory and field.

Local	*Megastigmus transvaalensis* progeny			
	Females	Males	Sex ratio	*P*-valor
Laboratory (drupes)	28.64	39.64	0.42	0.0181
Field (sticky traps)	10.03	116.44	0.08	0.1684

## Discussion

Yellow sticky traps are efficient to monitor *M*. *transvaalensis* in an ecological restoration area, similar to reports for *Psyllaephagus bliteus* Riek, 1962 (Hymenoptera: Encyrtidae) population fluctuations in a *Eucalyptus camaldulensis* plantation in São Paulo state^31^, Brazil, *Thaumastocoris peregrinus* Carpintero & Dellapé, 2006 (Hemiptera: Thaumastocoridae) in Rio Grande do Sul state, Brazil^32^ and *Leptocybe invasa* Fisher & La Salle, 2004 (Hymenoptera: Eulophidae) and of its parasitoid *Megastigmus* sp. (Hymenoptera: Torymidae) in the field in Dandeli, India^33^. These traps are the most attractive to Hymenoptera insects^34^ due to the yellow color resembling the bright leaves preferred by insects of this order as oviposition sites, as they can also be confused by appearance, which concerns the greater nitrogen quantity in the plant sap^34,35^.

*Megastigmus transvaalensis* adult peaks, from early May to September 2015, coincided with minimum and maximum temperatures of around 17 and 23 °C, respectively, similar to the increase in the *P*. *bliteus* adult numbers caught in fall with minimum and maximum temperatures^31^. This inverse correlation of the *M*. *transvaalensis* populations with temperature can be explained by the direct effect on the development of this insect, as reported for *L*. *invasa*, with high survival at lower temperatures^36^. Temperature decreases reduce metabolic rate, embryonic development, larva and pupa stages and affect insect behavior^37^, being pecilothermic organisms that are sensitive to temperature changes and thermal fluctuations^38^.

The lack of impact of relative humidity on the *M*. *transvaalensis* population fluctuation suggests that the mean humidity remained stable during the study period, since variations in this parameter affected the fecundity, longevity, parasitism and progeny of *Pediobius furvus* Gahan, 1928 (Hymenoptera: Eulophidae)^39^ in Kenya and *Cotesia flavipes* Cameron, 1891 (Hymenoptera: Braconidae) in Ethiopia, Africa^40^.

The inverse correlation of *M*. *transvaalensis* adult numbers captured with precipitation was similar to that reported for the decrease of *Trichogramma* (Hymenoptera: Trichogrammatidae) species numbers collected with increasing precipitation in Piracicaba, São Paulo state, Brazil^41^. This may be related to environmental resistance with physical and biological factors reducing insect population growth and climatic variables such as temperature and seasonal rainfall affecting these population structures^42^. The precipitation impact on *M*. *transvaalensis* is due to the mechanical control of populations of these insects, regardless of their population density^43^.

The *M*. *transvaalensis* population peak coincided with the highest number of plants with drupe (n = 13) of *S*. *terebinthifolia*, showing that this may be related to this host development^44^. This is similar to the higher activity of *Oomyzus sokolowskii* Kurdjumov, 1912 (Hymenoptera: Eulophidae) with increasing host numbers. The absence of viable hosts for parasitism drives the insects to search for longer periods for adequate hosts^45^, that is, the *M*. *transvaalensis* population increase depends on food availability in the field.

The lack of correlation between *M*. *transvaalensis* adult numbers caught and damaged *S*. *terebinthifolia* drupes is due to these individuals emerging a few months after oviposition^23^ on the mature drupe that remained on the trees. The diapause period of *Megastigmus* wasps varies depending on the host and the number of adults emerged on food availability^46^. Hymenoptera parasitoids have different strategies to avoid adverse environmental conditions^47^, with variations in the development of immature stages, adult stage duration, male and female maturation and diapause^28^.

The lack of effect of *S*. *terebinthifolia* fruiting on the *M*. *transvaalensis* adult numbers collected in the field during the population peak of this insect (May 20, 2015 with 927 insects), when the damage to the drupes collected was lower, reinforces the hypothesis that adults of this insect emerged from the drupes present on the trees or from previous fruits fallen on the ground. Damage in September 2014 may be related to previous flowering and fruiting periods during February to April of the same year, and the estimate of *M*. *transvaalensis* damage may have only been based on individuals who emerged from previous fruiting, since this insect presents diapause. However, *S*. *terebinthifolia* may also flower from October to December^48^, explaining damage during November and December 2014 through February 2015, with the end of one cycle and the beginning of another.

A *M*. *transvaalensis* generation was observed at 12 months at the beginning of the evaluations in 2014, when drupes present on the plants were from previous fruiting. Therefore, there was a single flowering and complete fruiting period, which was observed in 2015 and that can be explained by the flowering and fruiting differing between plants and localities, due to the wide geographic distribution and the morphological characteristics of each individual present^48^. The lack of *S*. *terebinthifolia* drupe production in the final evaluation period in the 19 trees selected may have affected the emergence of subsequent *M*. *transvaalensis* generations, since this insect may produce more than one generation per year according to flower and fruit production of this plant^24^. *Megastigmus* sp. presented three generations from May to November corresponding to spring, summer and autumn in the province of Adana, southern Turkey^49^.

Variability in the number of drupes damaged by *M*. *transvaalensis*, from zero to 36.3% and on *S*. *terebinthifolia* plants, 73.4%, is similar to that of this species with 1–55% in São Paulo state, Brazil, with a higher number of *S*. *terebinthifolia* plants in urban areas explaining that of damaged drupes (35.0 ± 15.8%) compared to restoration areas (15.8 ± 8.4%)^22^. Environmental conditions, age of the individuals sampled and *S*. *terebinthifolia* drupe production may explain damage variations between trees of this plant^24^.

The highest parasitism rate (36.3%) occurred during the spring 2014, under natural environmental conditions which presented temperatures of 16.7 to 27.6 °C, may be related to the previous *S*. *terebinthifolia* flowering periods, during October to November and February to April, which varied depending on the region as reported for this plant cultivated in the Botanical Garden of the Institute of Biosciences, Botucatu, São Paulo state, Brazil^50^. In Florida, USA, the parasitism peak was 31 and 76% during winter and spring, respectively, with temperatures different from those in Brazil^24^. In Argentina, *M*. *transvaalensis* parasitism on *S*. *molle* drupes of 6.29 to 26.84% occurred during autumn and winter fruiting^51^. In South Africa, this wasp damaged *S*. *molle* drupes throughout the entire summer rainy season^52^. Periods of greater parasitism and differences between sites may be related to *S*. *terebinthifolia* phenology. That is, periods of flowering and fruiting influence food availability and, consequently, the incidence of the wasp. These factors may affect the incidence of *M*. *transvaalensis*, similar to reports of significant variation in damage between trees and sites in Sorocaba, São Paulo state, Brazil^22^.

The greater emergence of *M*. *transvaalensis* females in the laboratory at 25 ± 2 °C, relative humidity of 60 ± 12% and photoperiod of 12:12 h (day: night) may be due to the controlled conditions. In the field, *S*. *terebinthifolia* drupes were naturally exposed, but with the emergence of *M*. *transvaalensis*, only when the conditions were favorable to this wasp. *Megastigmus transvaalensis* females can control their offspring sex at oviposition^53,54^ through environmental stimuli such as abiotic factors^55,56^. In addition, the greater number of males, captured on the sticky traps in the field than in the laboratory in all evaluations, can be attributed to arrhenotokous parthenogenesis^57^. The high or low sex ratio may be a response to environmental factors such as temperature and relative humidity with arrhenotokous parthenogenesis, Hymenoptera characteristics and linked to host size and age^58,59^. The sex ratio may vary with temperature as reported for *Trichogramma pretiosum* Riley, 1879 (Hymenoptera: Trichogrammatidae), with a higher female numbers at temperatures below 30 °C^60^. *Megastigmus* sp. sex ratio in laboratory was close to 1: 1 between 23 to 31 °C^49^.

Variations in body length, coloration, presence or absence of wing spots and size of the abdomen of *M*. *transvaalensis* males is similar to that reported for those emerged from *S*. *polygamus* drupes in Chile^20^. Chalcidoidea pigmentation varies between species of this superfamily and between sites and hosts, as reported for *Megastigmus dorsalis* Fabricius, 1978 (Hymenoptera: Torymidae) in Jordan^61^.

## Conclusions

The number of *M*. *transvaalensis* individuals was negatively correlated with maximum and minimum temperatures and precipitation, presenting population peaks at the end of May 2015. The parasitism rate by *M*. *transvaalensis* in *S*. *terebinthifolia* drupes ranged from zero to 36.3%, in field conditions. *Megastigmus transvaalensis* sex ratio was higher in the laboratory (0.42) than in the field (0.08). *Megastigmus transvaalensis* can be monitored with yellow sticky traps and males of this exotic wasp presented phenotypic variation in Brazil.

## Material and Methods

### Study sites

The study was carried out in an ecological restoration area near Semideciduous Seasonal Forest (SSF) fragments in an experimental area of the Federal University of São Carlos, Campus Sorocaba, São Paulo state, Brazil (23°34′S and 47°31′W) with average altitude of 580 m and annual precipitation of 1,311.2 mm from August 29, 2014 to September 9, 2015. *Schinus terebinthifolia* is referred to in the early stages of this type of forest according to Resolution of the National Environmental Council (CONAMA) of the Brazilian Ministry of Environment n° 392 of June 25, 2007. The climate of the region is of type “Cfa” (temperate humid with summer), according to the climatic classification of Köppen-Geiger^62^, with average annual temperature of 21.4 °C, maximum of 30.1 °C and minimum of 12.2 °C. The predominant soil type is Red Latosol^63^.

### *Megastigmus transvaalensis* adult sampling with sticky traps

The adult population survey of *M*. *transvaalensis* was carried out on 19 randomly selected, identified, georeferenced *S*. *terebinthifolia* plants with diameter at breast height (DBH) and tree height measured. *Megastigmus transvaalensis* was collected with traps consisting of yellow plastic cards (12 cm long x 10 cm wide) with adhesive on both sides and a capture area of 100 cm^2^ each^31^, discounting the area for the card identification. Each trap was installed on an *S*. *terebinthifolia* plant, attached to a plastic coated wire and fixed with a string at an approximate height of 2.50 meters.

Twenty-seven collections were performed approximately every 15 days when the traps were replaced with new ones, wrapped in transparent plastic film to avoid damaging the insects captured and placed into paper bags identified with the tree number and collection date. The traps were sent to the laboratory and stored at 0 °C until the *M*. *transvaalensis* adults were counted. Males and females of this wasp on both sides of the traps were counted using a stereoscopic microscope (Leica Microsystems TL3000 Ergo, Wetzlar, Germany) with 10X. The maximum and minimum temperature (°C), relative humidity (%) and precipitation (mm) values were obtained from the Meteorological Database for Education and Research - BDMEP Station N° 83851, Sorocaba, São Paulo state, Brazil (23° 29′ S and 47° 26′ W and 645 m altitude) of the National Institute of Meteorology (INMET). Readings were taken daily and the average maximum and minimum temperature, average relative humidity and accumulated precipitation, by evaluation date, were used.

### *Schinus terebinthifolia* drupe sampling

Branches with *S*. *terebinthifolia* drupes were collected at random in the middle third of the 19 plants (three branches per tree) with a pruning shear connected to a 3-meter long aluminum pole. These branches were collected, packed into paper bags when the yellow traps were collected and replaced, taken to the laboratory and stored in a Biochemical Oxygen Demand (BOD) incubator at 18 ± 2 °C to reduce the drying rate of leaves and drupes.

One hundred *S*. *terebinthifolia* drupes were chosen at random from three branches per sample point (tree) to calculate the *M*. *transvaalensis* parasitism rate. The total number of vesicular outflow holes (after the emergence of *M*. *transvaalensis* adults) was also counted under a stereoscopic microscope with 10X.

### *Megastigmus transvaalensis* sex ratio in laboratory and field

Twenty *S*. *terebinthifolia* drupes were randomly chosen from three branches/tree/evaluation and placed into 1,300 ml plastic containers with a lid, labeled and stored in BOD at 25 ± 2 °C, relative humidity of 60 ± 12% and photoperiod of 12:12 h (day: night) in the laboratory. *Megastigmus transvaalensis* males and females that emerged after approximately 15 days of incubation were counted, with females identified by the presence of the ovipositor at the extremity of the abdomen^23^. The *M*. *transvaalensis* sex ratio, sampled in the *S*. *terebinthifolia* drupes and those caught in the sticky traps were calculated.

### Data analysis

#### Megastigmus transvaalensis adult number variation

The number of insects captured in the sticky traps was subjected to multiple linear regression analysis^64^ using the meteorological variables data and the number of drupes damaged.

Statistical analyses were performed with the R Studio® program at a significance level of 5% in variance analysis (ANOVA) and input of the variables in the regression model. The variation coefficient was tested between trees to determine damage levels between them. The data were standardized for base 10 logarithm to reduce/equalize the value range and aid in the interpretation. This transformation was performed to evaluate the variance homogeneity and data distribution normality. A correlation matrix between the variables was generated and the data submitted to Tukey range test^65^. Shapiro-Wilk (W) normality test was used to verify if the dataset had normal distribution^66^, if there were differences between the means and if the factors could influence the dependent variable. The homogeneity of the variances was evaluated with the Levene test^67^.

The relationship between the data set variables and the trend visualization of the set, that is, the reduction or elimination of the number of variables, was verified by main component analysis (PCA). The eigenvalues, associated with a component or factor in descending order versus the number of the component or factor, were displayed in a scree plot graph of the data set with the broken-stick model^68^.

The multiple linear regression analysis was performed with the scores obtained from the PC1 and PC2 axes, adopting the model: *Yi* = *β*0 + *β*1*xi* + *€i*, to *i* = 1, … *n*.

The explanatory variables with the greatest contribution or predictive power were calculated with the significant axis for regression analysis PC1 (*P* = 0.0052) and the adjustment statistics of the model.

#### Schinus terebinthifolia drupe parasitism rate by Megastigmus transvaalensis

The *S*. *terebinthifolia* drupe parasitism percentage was calculated including those presenting *M*. *transvaalensis* outlet holes categorized as damaged (DD) and those without such holes as undamaged (ND). The difference between the damaged drupe values per evaluation was verified by ANOVA.

#### Megastigmus transvaalensis sex ratio

The sex ratio (RS) of *M*. *transvaalensis* was calculated (RS = females number ÷ insects number), submitted to variance analysis (ANOVA) and compared using the F test with 5% probability.
